# Epidermal growth factor receptor activity is elevated in glioma cancer stem cells and is required to maintain chemotherapy and radiation resistance

**DOI:** 10.18632/oncotarget.19868

**Published:** 2017-08-03

**Authors:** Lisa Y. Pang, Lauren Saunders, David J. Argyle

**Affiliations:** ^1^ Royal (Dick) School of Veterinary Studies and Roslin Institute, The University of Edinburgh, Easter Bush, Midlothian, EH25 9RG, Scotland

**Keywords:** glioma, cancer stem cells, comparative oncology, EGFR, radiation

## Abstract

Glioblastoma remains among the most aggressive of all human and canine malignancies, displaying high mortality rates and limited treatment options. We propose that given the similarities between canine and human gliomas, such as incidence of occurrence, histopathology, molecular characteristics, and response to therapy, that canine gliomas are a natural model of the human disease. A range of human and canine tumours have been shown to harbor specific subpopulations of cells with stem cell-like properties that initiate and maintain neoplasticity while resisting conventional therapies. Here, we show that both canine and human glioma cell lines contain a small population of cancer stem cells (CSCs), and by molecular profiling highlight the important role of the epidermal growth factor receptor (EGFR) pathway in canine CSCs. EGFR signaling is crucial in the regulation of cancer cell proliferation, migration and survival. To date EGFR-targeted interventions alone have been largely ineffective. Our findings confirm that specifically inhibiting EGFR signaling alone has no significant effect on the viability of CSCs. However inhibition of EGFR did enhance the chemo- and radio-sensitivity of both canine and human glioma CSCs, enabling this resistant, tumourigenic population of cells to be effectively targeted by conventional therapies.

## INTRODUCTION

Malignant gliomas are an aggressive form of brain cancer, in both humans and dogs, with a poor prognosis despite surgery, chemotherapy and ionising radiation [1–3]. In humans, the median survival time after diagnosis of glioblastoma multiforme (GBM), the most common form of malignant glioma, is 15 months [4]. There is a clear need for more effective strategies for the treatment of GBM, but many drug trials fail because the pre-clinical animal models do not sufficiently represent the human disease [5, 6]. The size and structure of the dog's brain, histopathology and molecular characteristics of canine GBM, as well as the presence of the intact immune system, all support canine brain tumours as naturally occurring models of human glioma [5, 7]. This comparative approach may yield translational advancement for novel diagnostic, drug and therapeutic studies.

In both human and veterinary medicine radiotherapy is a key treatment modality for brain tumours, but the efficacy is limited by radioresistance [8]. Recently, it has been established that in many types of cancer the bulk of the cells that make up the tumour are derived from a small population of cancer stem cells (CSCs) [9]. Glioma CSCs share characteristics with normal stem cells such as expression of neural stem cell markers, properties of self-renewal, extensive proliferation, and the ability to differentiate into more mature neural lineages. These cells have also been assigned a role in tumour angiogenesis and treatment resistance, and upon intercranial transplantation onto immunocompromised mice can successfully seed new tumours [10–15]. Thus, CSCs are likely to be responsible for initiation, progression and relapse of malignant gliomas, as conventional therapies that target the bulk of the tumour will fail to cure the disease because the CSCs will be unaffected and are able to recapitulate the tumour. Therefore it is imperative to target CSCs in the treatment of this disease.

There are genetic aberrations associated with GBM; amplification of epidermal growth factor receptor (EGFR) is a frequent finding that has been described in 40-50% of all human glioblastomas [16–18]. EGFR is a tyrosine kinase that is activated by ligand binding, thereby inducing receptor dimerization and auto-phosphorylation of key tyrosine residues. EGFR signaling affects many cellular events including cell survival, proliferation, differentiation, metabolism, and migration [7, 19]. In response to irradiation, EGFR promotes intracellular downstream signaling involving, among others, mitogen-activated protein kinase (MAPK) and phosphatidylinositol 3-kinase (PI3K), these pathways mediate cell survival and radio-resistance [20, 21]. EGFR also directly interacts with and enhances the activity of DNA-PK. DNA-PK plays a central role in non-homologous end joining double strand break repair [22, 23]. Activation of EGFR thereby exerts a protective function against DNA damage and promotes tumour cell survival.

In this study, we isolated and characterised CSCs from a canine and a human glioma cell line and showed that these cells express stem cell markers; are more invasive; more tumourigenic and more resistant to the cytotoxic effects of chemotherapy and ionising radiation, in comparison to their non-stem cell counterparts. Global transcriptional analysis of these CSCs revealed that they have a distinct gene expression profile consisting of over 10,000 significant differences, compared to non-CSCs. Moreover, a number of the most upregulated genes in CSCs are activators of EGFR. Concurrently we showed that CSCs have enhanced basal activation of the EGFR pathway that can be inhibited by the EGFR tyrosine kinase inhibitor, gefitinib. Our findings demonstrated that gefitinib treatment enhanced radio-sensitivity of both canine and human glioma CSCs, enabling this resistant, tumourigenic population of cells to be effectively targeted by ionising radiation. This data further validates canine glioma as a model for the human disease supporting the development of new therapeutic strategies in both human and veterinary medicine.

## RESULTS

### Glioma stem cells express stem cell markers, increased invasiveness and increased tumourigenicity

**Figure 1:**
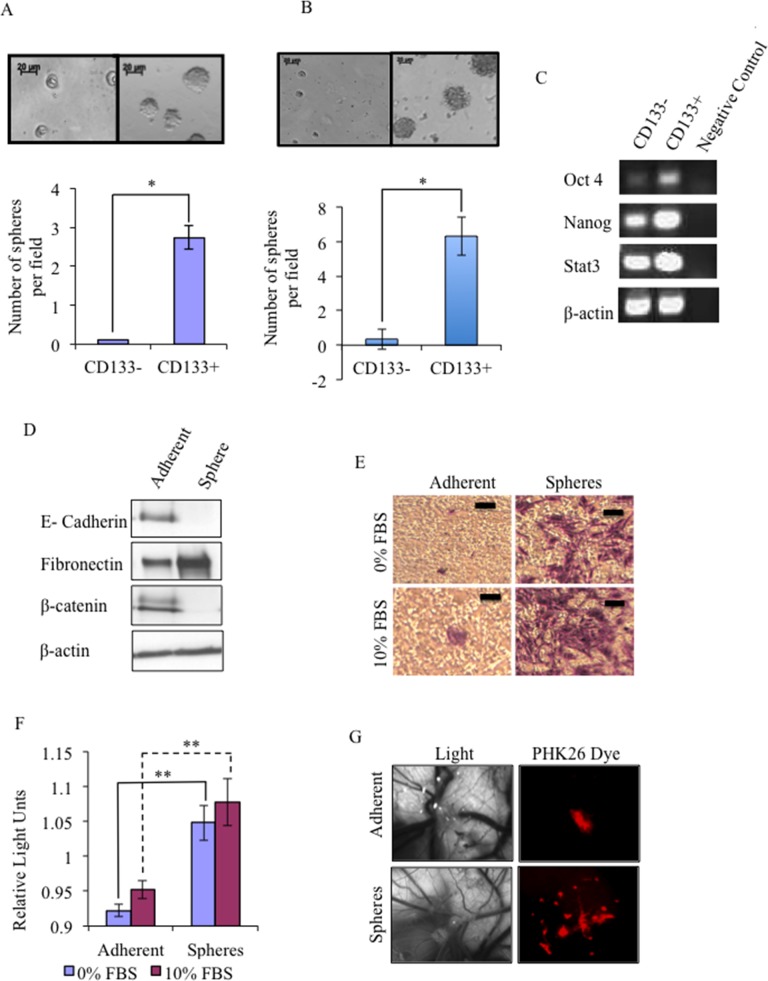
Isolation and characterisation of glioma cancer stem cells (CSCs) A small population of CD133+ cells exists in canine glioma J3T cell line **(A)** and the human glioma LN18 cell line **(B)** and readily form spheres compared to CD133- cells. Data are representative of three independent experiments ±SD (*p < 0.001). Images were taken at 40x magnification. **(C)** Reverse transcriptase (RT)-PCR analysis of embryonic stem cell markers: *Nanog*, *Oct4*, *STAT3*, and *β-actin* gene expression levels of J3T CD133+ and CD133- cells. **(D)** Western blots analysis of cell lysates derived from J3T adherent cells and spheres for markers of EMT: E-cadherin, fibronectin, β-catenin, with β-actin as a loading control. 30 μg was loaded per lane **(E)** J3T CSCs show an increased invasive potential *in vitro*. Invasive ability of J3T adherent cells and spheres was analysed using a collagen based invasion assay. **(F)** Invading cells were quantified by measuring the optical density at 560 nm (** *p* < 0.005). **(G)** Glioma CSCs are enriched for higher tumourigenicity *in vivo*. Disassociated J3T spheres and adherent cells were inoculated directly onto the chorioallantoic membrane of a chicken embryo at day 7 of development. All cells were fluorescently labelled and imaged 3 days after inoculation.

In this study we have utilised an established serum-free cell culture system that favours the proliferation of undifferentiated cells thereby enriching for CSCs, which grow as spheres [13]. Expression of the stem cell marker CD133 was characterised by magnetic cell sorting [13]. Cells from the canine glioma cell line J3T and the human glioma cell line LN18 contain a small subpopulation of CD133+ cells (1.17 ±0.58 % (*n* = 8) and 2.07 ±0.43 % (*n* = 4), respectively), that are significantly better at forming spheres (Figure 1A and 1B), and express higher levels of the embryonic stem cells markers *Oct4*, *Nanog* and *STAT3* (Figure 1C) than CD133- cells (similar results were obtained for LN18 CSC, data not shown).

The metastatic potential of CSCs, which is the ability of a CSC to migrate from the tumour microenvironment and subsequently invade and attach at a secondary site, is proposed to be mediated by the epithelial-to-mesenchymal transition (EMT) [24, 25]. Here we show that J3T spheres have a mesenchymal phenotype, whereby expression of E-Cadherin and β-catenin was significantly decreased, and that of Fibronectin was significantly increased compared to adherent cells (Figure 1D), and that CSCs are much more invasive *in vitro* (Figure 1E and 1F) (similar results were obtained for LN18 CSC, data not shown). To determine if spheres were more likely to form tumours *in vivo* than adherent cells, we utilised the chicken embryo chorioallantoic membrane (CAM) model: the CAMs of day 7 chicks were inoculated with either fluorescently labelled dissociated spheres or adherent cells. At day 10 of development 3-dimensional tumours were visible in 100% of membranes inoculated with dissociated spheres but not adherent cells. These micro-tumours were visualized under the fluorescence microscope; sphere cells were brightly fluorescent and had radiated out from the 3-dimensional tumour growths, invading the surrounding blood vessels of the CAM. In contrast, adherent cells were localised to the initial site of inoculation (Figure 1G). Therefore CSCs have a greater *in vivo* tumourigenic capacity than non-CSCs cells.

### CSCs exhibit greater resistance to radiation-induced cytotoxicity

**Figure 2:**
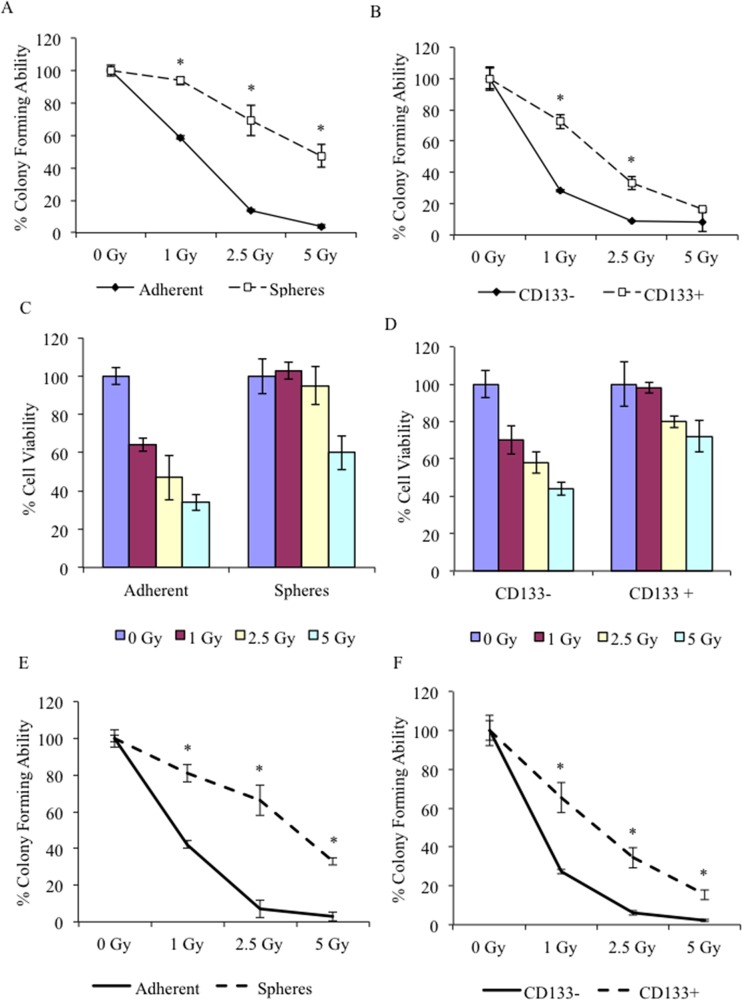
CSCs are resistant to radiation treatment Analysis of colony forming ability was assayed after J3T adherent cells and spheres **(A)**, and CD133 sorted cells **(B)** were treated with increasing doses of ionising radiation. Cell viability of J3T adherent and spheres **(C)** and CD133 sorted cells **(D)** was assayed 48 hours after treatment. Analysis of colony forming ability was assayed after LN18 adherent cells and spheres **(E)**, and CD133 sorted cells **(F)** were treated with increasing dose of ionising radiation. (* *p* < 0.005).

To determine whether spheres cells preferentially survive after treatment with external beam radiation, spheres derived from J3T and LN18 cell lines, were disassociated into single cells and treated with increasing doses of ionising radiation. Clonogenic survival was determined: J3T and LN18 spheres demonstrated a significantly increased resistance to radiation-induced replicative cell death compared to non-CSC adherent cells (Figure 2A and 2E, respectively). Similar results were obtained when CSCs were isolated by expression of CD133 (Figure 2B and 2F, respectively). Cell viability was assayed 48 hours after treatment: J3T non-CSCs showed a dose-dependent decrease in cell viability whereas CSCs were inherently resistant to the cytotoxic effect of radiation (Figure 2C and 2D), and therefore in a physiological setting may contribute to tumour repopulation.

### Treatment of CSCs with doxorubicin increases the size of the CSC population and highlights defects in activation of p53

**Figure 3:**
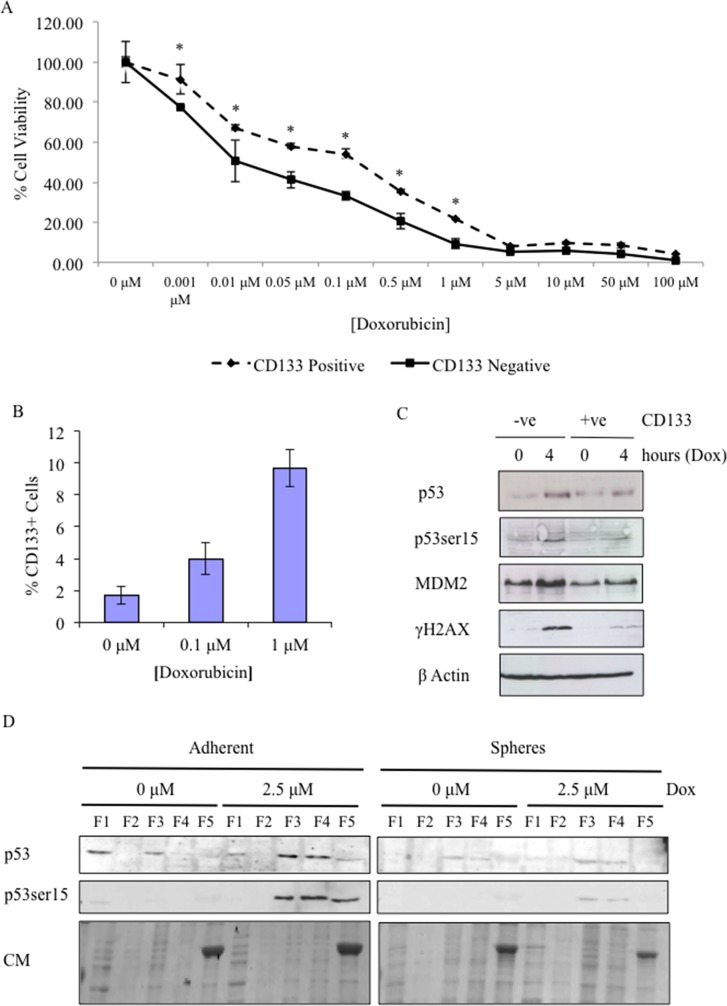
Doxorubicin treatment enriches CSCs subpopulation and highlights that in CSCs p53 activity is attenuated and aberrantly localised within the cell **(A)** Cell viability of MACS-sorted J3T CD133+ and CD133- cells was assayed 48 hours after treatment with increasing doses of doxorubicin (* *p* < 0.005). **(B)** J3T cells were treated with the indicated doses of doxorubicin and the percentage CD133+ cells was determined 24 hours post-treatment. **(C)** CD133+ and CD133- cells were treated with doxorubicin and harvested 4 hours after treatment. Cell lysates were probed for the expression of phosphorylation of p53 at serine-15, MDM2, γH2AX and β-actin as a loading control. 30 μg was loaded per lane. **(D)** The subcellular localisation of p53, in J3T adherent cells and spheres, was determined 4 hours post-treatment with the indicated doses of doxorubicin by western blotting. Proteins were extracted according to their subcellular localisation: F1, cytosolic; F2, membranes/organelles; F3, nucleus; F4, nucleus; F5, cytoskeleton. 30 μg was loaded per lane. Coomassie staining (CM) confirmed that protein expression profiles from each fraction were distinct, 5 μg was loaded per lane.

Similarly, J3T CD133+ cells were resistant to the cytotoxic effect of the chemotherapy drug, doxorubicin. Doxorubicin is an anti-tumour antibiotic DNA damaging agent and is commonly used in veterinary and human cancer chemotherapy protocols. Here, CD133+ and CD133- cells were treated with increasing concentrations of doxorubicin and cell viability was assayed 48 hours after treatment. CD133+ cells demonstrated significantly increased resistance to doxorubicin induced cell death compared to CD133- cells (Figure 3A). Doxorubicin treatment also increased the size of the CD133+ population in a dose dependent manner (Figure 3B), further supporting the finding that CSCs are resistant to doxorubicin treatment. To determine if resistance of CSCs to DNA damaging agents was due to defects in the p53 pathway, we treated CD133 sorted cells with doxorubicin and by western blotting analysed protein expression of key members of the pathway. In response to doxorubicin, CD133- cells showed a transient increase in p53 levels and phosphorylation of p53 serine-15, an ATM target that is associated with activation of p53 transcriptional activity [26], and of the p53 transcriptional target MDM2 at 4 hours post-treatment (Figure 3C). Levels of γH2AX, an ATM target and a marker of DNA double strand breaks [27], similarly increased 4 hours post-treatment in CD133- cells (Figure 3C), whereas, in CD133+ cells induction of γH2AX, p53, phosphorylation of p53 serine-15, and induction of MDM2 was markedly reduced (Figure 3C). The primary role of p53 is as a stress-activated transcription factor therefore reduced p53 activity in CSCs may be linked to p53 protein localization [28]. In untreated non-CSC adherent cells, p53 protein was detected at a low level and was located in the cytosolic and nuclear fractions, whereas in untreated CSCs p53 protein levels were relatively low and associated with the nuclear fractions (Figure 3D). Upon DNA damage, the p53 protein levels in adherent cells increased in both the nuclear fractions, whereas in CSCs p53 protein levels and subcellular localisation remain unchanged (Figure 3D). Similarly, phosphorylation of p53 at serine-15 was undetectable in untreated adherent and sphere cells, but upon DNA damage a high level of phosphorylation of p53 at serine-15 in the nuclear fractions of adherent cells was detectable, whereas there was a low level of phosphorylation of p53 at serine-15 detected in CSCs (Figure 3D). The total protein content of each fraction was evaluated qualitatively by coomassie staining (Figure 3D).

### Global analysis of gene expression reveals significant differences in the molecular profiles of CSCs and non-CSCs

**Figure 4:**
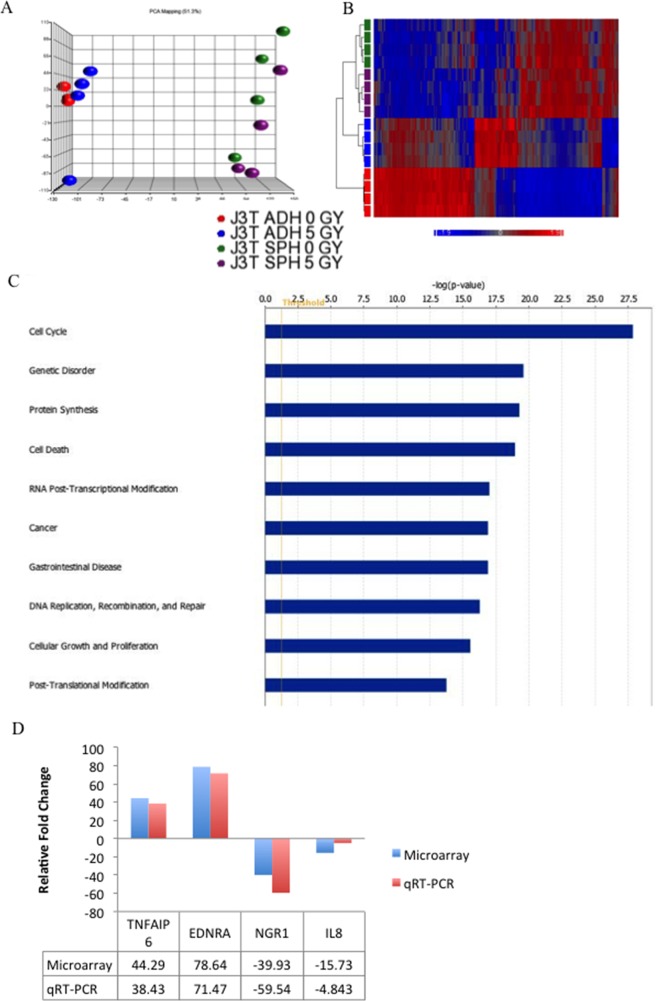
Gene expression analysis of canine glioma stem cells **(A)** A three-dimensional representation of a principle component analysis of expression microarray data derived from untreated J3T adherent cells (0 Gy), irradiated adherent cells (5Gy), untreated spheres (0 Gy) and irradiated spheres (5 Gy). **(B)** Hierarchical clustering analysis of the expression data (cut off *p*-value of 0.005). Expression values are represented by colours: blue squares represent low-expressed genes, red squares represent high-expressed genes. **(C)** Biological process analysis of differentially expressed genes in J3T spheres compared to adherent cells (FDR = 0.005). **(D)** Validation of microarray with qRT-PCR.

We performed gene expression profiling of J3T CSCs and non-CSCs, minus (0 Gy) or plus (5 Gy) external beam radiation, using the Affymetrix GeneChip® Canine 2.0 Array. Untreated CSCs, represented by sphere cells, differentially expressed (i.e. up- or down regulated >2-fold with a false discovery rate (FDR) of 0.05) 15,193 genes compared to non-CSCs. To obtain a manageable number of gene differences, the FDR was decreased to 0.005. Under these parameters, 10,358 genes were differentially expressed in CSCs compared to non-CSCs. A similar number of gene expression changes were observed between CSCs and non-CSCs post-irradiation treatment (Table 1). Interestingly, radiation induced more mRNA changes in non-CSCs (265) compared to CSCs (45). Principle component analysis shows a distinct separation of CSC and non-CSC populations (Figure 4A) and the heatmap shows that non-irradiated and irradiated CSCs cluster more closely than with non-irradiated and irradiated non-CSCs (Figure 4B). Further pathway analysis showed that the differential expression profile of non-irradiated CSCs encompassed genes involved in a variety of biological processes and diseases including cell cycle regulation, protein synthesis, cell growth, proliferation, DNA repair, apoptosis, and cancer (Figure 4C). Significantly, genetic disorder and cancer were the top diseases identified in the analysis (Table 2), indicating that gene expression profiles associated with cancer are more prevalent in the CSC population than the non-CSCs. The top ten upregulated genes in CSCs compared to non-CSCs are shown in Table 3. Similar results were obtained for irradiated CSCs compared to irradiated non-CSCs (data not shown). Validation of the microarray was carried out by qRT-PCR (Figure 4D).

**Table 1:** Primer sequences for the amplification of RT-PCR products from canine cells

Gene	Forward primer (5′-3′)	Reverse primer (5′-3′)	Product size
*Oct4*	CTCTGCAGCCAATCAACCACAA	GGAGAGGGGGATGAGAAGTACAAT	237 bp
*Nanog*	CTATAGAGGAGAGCACAGTGAAG	GTTCGGATCTACTTTAGAGTGAGG	160 bp
*STAT3*	GTGGAGAAGGACATCAGCGGTAA	AACTTGGTCTTCAGGTATGGGGC	250 bp
*β-Actin*	CATGTTTGAGACCTTCAACACCC	GCCATCTCTTGCTCGAAGTCCAG	229 bp

**Table 2:** Primer sequences for the amplification of qRT-PCR products from canine cell lines

Gene	Forward primer (5′-3′)	Reverse primer (5′-3′)
*TNFAIP6*	ACGGTTTTGTGGGAAGGTACT	TTTGGAAACCTCCCGCTGTC
*EDNRA*	GAATTACTTAGTTTCTTGCGTCTCA	GGACTGGTAACAGCAACAGC
*NGR1*	CTGGTGATCGCTGCCAAAAC	AGAGCTCCTCCGCTTCCATA
*IL8*	TGTTGCTCTCTTGGCAGCTT	CGGATCTTGTTTCTCAGCCTTCTT
*MRPS7*	AGTGCAGGGAGAAGAAGCAC	CAGCAGCTCGTGTGACAACT
*GAPDH*	GGGAAGATGTGGCGTGAC	GAAGGCCATGCCAGTGAG

**Table 3:** The number of differentially expressed genes between the indicated cell populations of J3T spheres (SPH) and adherent (ADH) cells

	p = 0.05	p = 0.005
J3T SPH V ADH (0 Gy)	15,193	10,358
J3T SPH V ADH (5 Gy)	13,634	8,841
J3T SPH (5 Gy) V SPH (0 Gy)	239	45
J3T ADH (5 Gy) V ADH (0 Gy)	1338	265

### EGFR is constitutively active in CSCs

**Figure 5:**
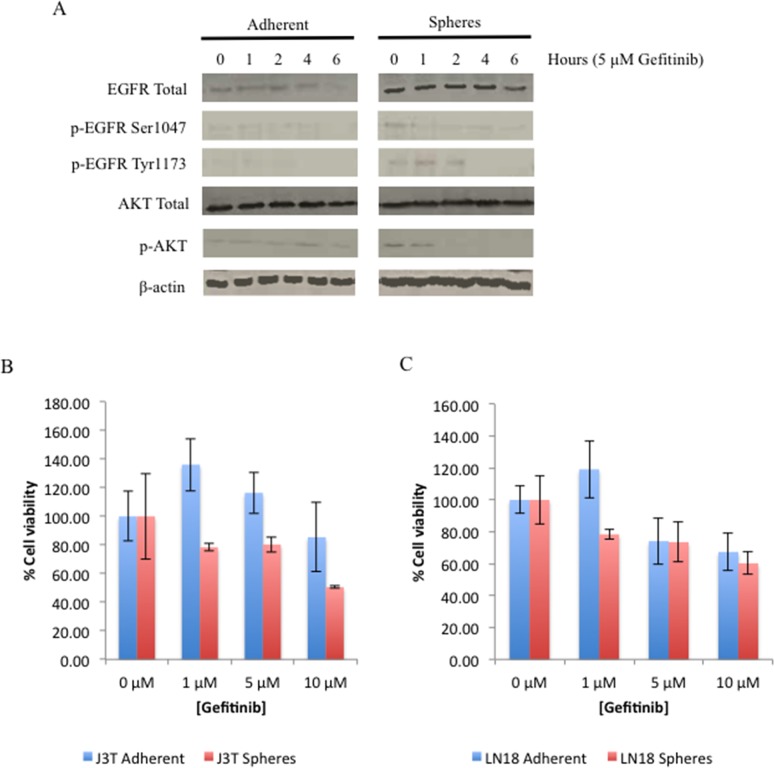
EGFR is constitutively active in CSCs **(A)** J3T adherent and sphere cells were treated with 5 μM gefitinib and harvested over the indicated time course. Expression of EGFR, phosphorylation of EGFR at serine-1047 and tyrosine-1173, AKT, phosphorylation of AKT, and β-actin as a loading control. 20 μg was loaded per lane. Comparison of the effect of increasing doses of gefitinib on proliferation of adherent and spheres of J3T **(B)** and LN18 cells **(C)**. Cells were treated with increasing doses of gefitinib and cell viability was assayed 48 hours post treatment.

Activators of the EGFR pathway, including EDNRA, CXCR7, IGFBP2 and EGR1, dominated the top 10 upregulated genes in CSCs compared to non-CSCs, as identified by microarray analysis. Although elevation of EGFR gene expression was not detected, CSCs do exhibit a higher basal level of EGFR protein that is constitutively phosphorylated at serine-1047 and tyrosine-1173. Constitutive phosphorylation of AKT, a downstream target of EGFR, indicates that the EGFR pathway is constitutively active in CSCs in contrast to non-CSCs (Figure 5A). To determine the effect of EGFR inhibition J3T adherent cells and spheres were treated with gefitinib, a selective inhibitor of the tyrosine kinase site in the catalytic domain of EGFR. In CSCs, gefitinib inhibited phosphorylation of EGFR at serine-1047 and tyrosine-1173, and inhibited activation of AKT. The total levels of EGFR and AKT were unaffected until 6 hours post-treatment where there is a decrease in EGFR levels in both cell populations. Non-CSCs showed no basal activation of the either the EGFR or AKT pathway (Figure 5A).

### Inhibition of EGFR enhanced CSCs chemosensitivity

Surprisingly, gefitinib treatment alone had no significant effect on the cell viability of both canine and human CSCs (Figure 5B and 5C, respectively), but enhanced the proliferation of non-CSCs until doses exceeding 5 μM were used, whereby cell viability, of all cell populations, decreased in a dose dependent manner. This was the dose of gefitinib selected for further experiments.

**Figure 6:**
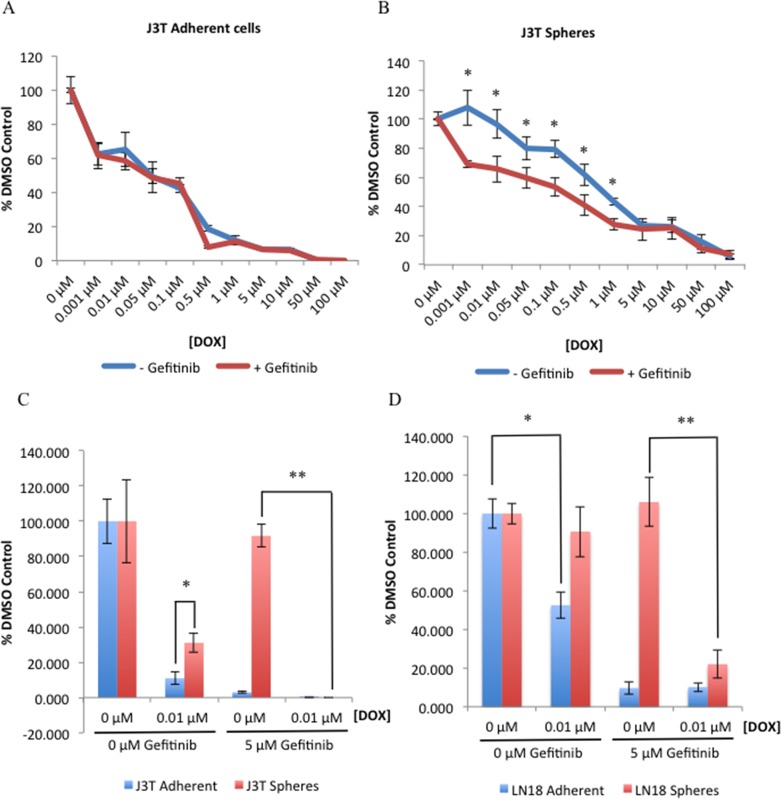
Gefitinib decreases CSC resistance to doxorubicin J3T adherent **(A)** and spheres **(B)** were pre-treated with either a DMSO control or gefitinib (5 μM) for 24 hours, incubated with the indicated doses of doxorubicin and cell viability was assayed 48 hours post-doxorubicin treatment. Colony forming ability of adherent and spheres derived from J3T **(C)** and LN18 **(D)** cell lines was assayed after cells had been pre-treated with either a DMSO control or gefitinib (5 μM) for 24 hours prior to doxorubicin treatment (* *p* < 0.005, ** *p* < 0.001).

To determine the effect of gefitinib on chemosensitivity, J3T adherent and sphere cells were pre-incubated with gefitinib for 24 hours prior to treatment with increasing doses of doxorubicin for 48 hours, before cell viability was analysed. Combinational gefitinib and doxorubicin treatment had no effect on adherent non-CSCs (Figure 6A). However, in CSCs inhibition of EGFR enhanced the sensitivity of these cells to the cytotoxic effects of doxorubicin compared to control (Figure 6B). To substantiate these findings we analysed the clonogenic survival of doxorubicin treated cells with and without gefitinib. J3T CSCs are inherently more resistant to doxorubicin than non-CSCs in the absence of EGFR inhibition, however gefitinib enhanced the chemosensitivity of CSCs to a level comparable to non-CSCs (Figure 6C). Interestingly, gefitinib alone reduced the colony forming ability of non-CSCs but had no effect on CSCs, until the cells were challenged with chemotherapy (Figure 6C). Similar results were obtained with human LN18 cells (Figure 6D).

### Inhibition of EGFR sensitises glioma CSCs to radiation-induced cytotoxicity

**Figure 7:**
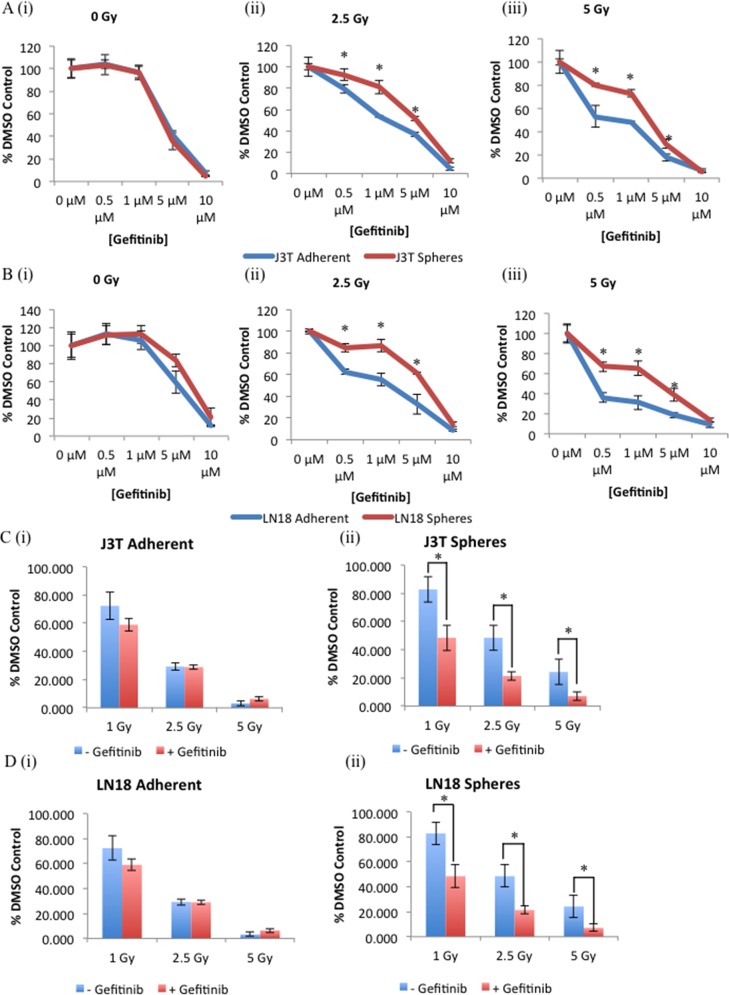
Inhibition of EGFR sensitises glioma CSCs to radiation-induced cytotoxicity J3T **(A)** and LN18 **(B)** adherent and spheres were treated with the indicated doses of gefitinib for 24 hours prior to treatment with either 0 Gy (i), 2.5 Gy (ii), or 5 Gy (iii), cell viability was assayed 48 hours post-irradiation treatment. Colony forming ability of adherent (i) and spheres (ii) derived from J3T **(C)** and LN18 **(D)** cell lines was assayed after cells had been pre-treated with either a DMSO control or gefitinib (5 μM) for 24 hours prior to treatment with indicated doses of irradiation (* *p* < 0.005).

As radiation treatment is the principal modality for treating glioma, we tested the combinational effect of EGFR inhibition and radiation on cell proliferation and clonogenic survival. Canine J3T (Figure 7A) and human LN18 (Figure 7B) adherent cells and spheres were treated with increasing doses of gefitinib (0 μM – 10 μM) and increasing doses of ionising radiation (0 Gy – 5 Gy). Gefitinib alone had no significant effect on the cell viability of either cell population (Figure 7A i) and 7B i)) until doses exceeding 5 μM were used. However, gefitinib in combination with ionizing radiation doses of 2.5 Gy (Figure 7A ii) and 7B ii)) and 5 Gy (Figure 7A iii) and 7B iii)), significantly increased the sensitivity of both CSCs and non-CSCs to radiation-induced cell death in a dose-dependent manner. Consistent with previous results, CSCs were more resistant to the effects of EGFR inhibition than non-CSCs ((Figure 7A, 7B ii) and iii)). Clonogenic survival analysis is a longer-term assay that measures cell reproductive death after treatment, and here shows that non-CSCs were unaffected by pre-treatment with gefitinib prior to radiation at the indicated doses (Figure 7C i) and 7D i)). Whereas inhibition of EGFR sensitised CSCs to the cytotoxic effects of increasing doses of ionising radiation (Figure 7C ii) and 7D ii)). These results indicate that combinational targeting of EGFR may be an effective method to overcome the intrinsic insensitivity to chemotherapy and radiation of CSCs.

## DISCUSSION

GBM, the most aggressive and lethal primary brain tumour in both humans and dogs, portends a poor prognosis despite surgery, chemotherapy, and radiation [2, 4, 5]. GBM is characterized by a high degree of heterogeneity that reflects the presence of multiple subpopulations of cancer cells within the same tissue [29, 30]. Recurrence of tumour growth is attributed to the presence of treatment-resistant CSCs [8]. Future targeting of these CSCs is therefore essential in the treatment of this disease. CSCs have been isolated from primary cases of human [8, 10–13, 15] and canine GBM [14].

To date there are no bona fide cell surface markers available to distinguish non-CSCs from CSCs. CD133, which is expressed on hematopoietic [31] and neural stem cells [32], is widely used as a CSC marker in several tumours, including GBM, where cell sorting for CD133 expression can enrich for cells with tumourigenic potential [12, 13]. However, conflicting findings have called into question the utilization of this marker in brain tumours: there is a high variability of CD133 expression in GBM (from 1% to 60%); and GBM stem cells not expressing CD133, which are able to self-renew and regenerate tumours in the xenotransplantation assay, have been identified [33–35]. A recent study showed that neurospheres are composed of CD133-positive and CD133-negative cells, and although CD133-negative cells do not express CD133 on the plasma membrane they do show diffuse cytoplasmic staining [36]. Furthermore, the subcellular localization of CD133 is plastic: shuttling between the cytoplasm and plasma membrane in response to cues from the microenvironment. Brescia *et al* (2013) concluded that CD133 is essential to the maintenance and tumourigenic potential of GBM CSCs [36]. Due to the ambiguity of CD133 as a universal marker of CSCs, we choose the neurosphere assay as a surrogate for *in vitro* study of GBM CSCs. In the present study, we have demonstrated that rare sub-populations of cancer cells exist in established canine and human glioma cell lines, which possess several distinct functional properties of CSCs, including expression of CD133, *in vitro* self-renewal, increased invasiveness, preferential expression of embryonic stem cell markers and markers of EMT, and *in vivo* tumourigenic capacity.

We have also shown that CSCs are inherently resistant to chemotherapy, and due to this, doxorubicin treatment enhances the size of the CSC pool. Interestingly, CSCs lacked activation of the stress-activated transcription factor p53 and phosphorylation of γH2AX, an ATM target and a marker of DNA double strand breaks [26, 27]. This is consistent with human and mouse embryonic stem cells that lack the cell cycle checkpoint between G1 and S-phase and do not senesce after DNA damage, due to an inactive p53 pathway, which is to avoid severe depletion of the functional stem cell pool. Subsequent studies have highlighted that over-expression of p53 in ESCs leads to a suppression of self-renewal and induction of differentiation, and in the context of reprogramming, expression of wild-type p53 constrains iPSC generation *in vitro* [37–39]. Given that CSCs could arise from either the accumulation of genetic insults in normal stem cells or by dedifferentiation of existing differentiated cells, and that p53 is both a driver of differentiation and a barrier of dedifferentiation, our findings contribute to evidence that inactivation of p53 is a stem cell trait.

Global analysis of gene expression showed vast differences between J3T CSCs and non-CSCs, with 10,358 significant differences. Comparisons of non-irradiated and irradiated CSCs revealed fewer significant differences (45), than that of non-CSCs (265), indicating that CSCs may indeed have a dampened down DNA damage response, reflected by reduced activity of ATM and p53. Data mining for biologically relevant targets highlighted that activators of the EGFR pathway, including EDNRA [40], CXCR7 [41, 42], IGFBP2 [43] and EGR1 [44], are overexpressed in CSCs compared to non-CSCs. Even though EGFR itself was not overexpressed we showed that EGFR was constitutively active in CSCs and that this can be attenuated by treatment with the small molecule tyrosine kinase inhibitor, gefitinib. In humans EGFR is a potent driver of oncogenesis; over-expressed in a range of tumours; and is associated with worse overall survival [18, 45]. Similarly in dogs, there is significantly greater expression of EGFR in GBMs compared to other canine inter-cranial brain tumours that have been documented [46, 47]. In both neural stem cells and glioma CSCs activated EGFR signaling increases proliferation, survival, migration and blocks differentiation [7]. Here we show that inhibition of EGFR signaling alone had no effect on the viability of CSCs, however it does sensitise CSCs to the cytotoxic effects of chemotherapy and ionising radiation. In head and neck cancer resistance of tumour cells to radiotherapy has been shown to increase proportionally with EGFR expression, and it has been proposed that up-regulation of EGFR is a mechanism employed by cancer cells to circumvent the cytotoxic effects of radiotherapy [48, 49].

The effect of EGFR-targeted therapy has been extensively tested in preclinical human glioma models [50–52]. Small molecule tyrosine kinase inhibitors, including erlotinib and gefitinib, are well established in clinical practice for the treatment of lung carcinomas, yet showed a lack of activity in glioma [52]. An alternative approach is to use antibodies directed against EGFR, including cetuximab, nimotuzumab, and panitumumab, that prevent the binding of EGFR ligands to the receptor. Although these antibodies are clinically relevant in the treatment of advanced colorectal carcinoma [53, 54] and metastatic breast cancer [55, 56], their implementation against intracranial neoplasms are challenging due to the presence of the blood-brain barrier, which may preclude the penetration of the antibody to all parts of the tumour. This has been mirrored in the treatment of glioma patients with pharmacological inhibitors of EGFR or blocking antibodies that fail to show signs of activity, indicating that inhibition of EGFR alone is insufficient [57–59]. It is interesting to speculate that termination of EGFR signaling may lead to the acquisition of compensatory additional genetic events, such as the expression of alternative tyrosine kinase receptors, and that disruption of converging signaling pathways may help overcome resistance to EGFR inhibitors.

Several studies have previously suggested a sensitizing effect of inhibition of EGFR to radiation [23, 60–63]. Clinical trials to explore the combination of radiation therapy with inhibition of EGFR in patients with newly diagnosed glioblastoma failed to show an increase in survival compared to historical controls [64, 65]. Patients were not selected for these trials on the basis of high expression of EGFR. However gene amplification or expression of wild-type EGFR or expression of a mutant form called EGFR variant III (EGFRvIII) is found in approximately 40-50% of all human gliomas [16]. EGFRvIII arises from an in-frame deletion of exons 2-7 from the extracellular domain, leading to a loss of ligand binding potential, constitutive activation of the receptor and resistance to gefitinib [66]. Rather than global overexpression of EGFR throughout the tumour, our data in the J3T cell line shows that a minority population of CSCs do not over-express of EGFR but do have constitutive activation of EGFR compared to the bulk of the tumour cells, that can be attenuated by treatment with gefitinib. Successful targeting of CSCs is of paramount importance as these cells have been suggested to be responsible for the treatment resistance and recapitulating tumour growth in GBM patients. Radiotherapy is currently the key treatment modality for GBM, but efficacy is limited by radio-resistance that may be mediated by CSCs. We have shown that CSCs were significantly more resistant to chemotherapy and radiation than non-CSCs, and that inhibition of EGFR, by gefitinib, can sensitise both human and canine glioma CSCs to the cytotoxic effects of doxorubicin and radiation. Our data indicates that inhibition of EGFR has a role as part of a multimodal therapy in overcoming resistance of CSCs to conventional therapies.

## MATERIALS AND METHODS

### Cell culture and sphere formation

The tumour cell lines used in this study were the canine glioma cell line J3T [67, 68], and human glioma cell line LN18 [69], both were obtained commercially (ATCC, Middlesex, UK). J3T cells were grown in Dulbecco's modified Eagle's medium (DMEM) (Invitrogen, Paisley, UK) supplemented with 10 % fetal bovine serum and 100 μg/ml streptomycin (Invitrogen, Paisley, UK). LN18 were grown in DMEM (Invitrogen, Paisley, UK) supplemented with 10 % fetal bovine serum and 100 μg/ml streptomycin.

For anchorage-independent culture, cells were plated as single cells in ultra-low attachment 6-well plates (Corning, CA, USA) at low cell density (1.5×10^4^ cells/ml). Cells were grown in serum-free conditional medium, which contained William's E Medium with GlutaMAX supplemented with putrescine (100 μM), sodium selenite (30 nM), transferrin (25 μg/ml), insulin (20 μg/ml) (Sigma Biochemicals, Dorset, UK), human recombinant bFGF (10 ng/ml) (Peprotech, NJ, USA). Additional bFGF (100 μg/ml) was added to the media every other day. All cell cultures were maintained at 37°C in a humidified CO_2_ incubator.

### Magnetic cell sorting

Cells were labelled with CD133 microbeads and sorted using the Miltenyi Biotec CD133 cell isolation kit according to the manufacturer's protocol (Miltenyi Biotec, Surrey, UK). Briefly, cells were resuspended in 300 μl PBS solution (pH 7.2, 0.5% BSA, 2 mM EDTA) per 10^8^ cells. Then blocking reagent FcR (100 μl/10^8^ cells; Miltenyi Biotec, Surrey, UK) and CD133 microbeads (100 μl/10^8^ cells) were added and mixed at 4°C for 30 minutes with rotation. Cells were washed in 20x volume with PBS solution. The pellet was resuspended in 500 μl PBS solution and added to a pre-washed magnetic separation (LS) column on the magnetic holder. The column was washed four times and the cells were collected as the negative fraction. The column was removed from the magnetic holder and the positive fraction was collected.

### Sphere forming efficiency

The sphere forming ability of CD133 sorted cells was determined by resuspending cells in serum-free conditional medium at a density of 20,000; 10,000; 5,000; or 2,000 cells/well in 6–well low adherence plates (Corning, CA, USA). All experiments were conducted in triplicate. Plates were maintained at 37°C in humidified CO_2_ incubator and were fed every other day. After 10 days colonies were counted under the microscope in 10 fields per well.

### Cytotoxic drug or radiation treatment

Cells were grown to 70% confluency before treating with indicated drug or stress. Cells were irradiated in culture medium using a Faxitron^®^ cabinet X-ray system, 43855D (Faxitron X-ray Corporation, Lincolnshire, IL, USA), at a central dose rate of 2 Gy/min. Cells were irradiated at the stated doses and harvested at the stated time points. Cells were treated with doxorubicin (Pharmacia/Pfizer, Sandwich, UK) or gefitinib (Tocris Bioscience, Bristol, UK), within the indicated dose range. All drugs were dissolved in dimethyl sulfoxide, and diluted in media immediately before use. Vehicle controls were included in all experiments.

### Analysis of cytotoxicity

Cells were trypsinised into single cells and seeded in quadruplet in opaque 96-well plates (Corning, CA, USA) at 500 cells/well. Serial dilutions of doxorubicin, gefitinib, or ionising radiation were added to the appropriate cells the following day or as indicated. Dose-response curves were generated 48 hours after exposure. Cytotoxicity was measured using the CellTiterGlo® Luminescent Cell Viability Assay (Promega, Madison, USA), which quantifies the number of viable cells in culture based on quantification of ATP present. Luminescence was recorded by luminometer (Viktor3, PerkinElmer, Massachusetts, USA). Data was averaged and normalized against the average signal of untreated/vehicle control treated samples.

### Colony formation assay

Cells were trypsinised into single cells and seeded at 500 cells/10 cm plate. The cells were treated with the indicated dose of doxorubicin or ionising radiation whilst in suspension. Plates were incubated at 37°C in humidified CO_2_ incubator until colonies were visible. Growth media was changed once a week. The colonies were fixed by incubating with ice-cold methanol for 5 minutes at room temperature. Colonies were stained with Giemsa stain (Invitrogen, Paisley, UK) according to the manufacturer's instruction. The total number of colonies was counted.

### Invasion assay

The cell invasion ability of isolated cells was determined using the QCM™ collagen-based cell invasion assay kit (Millipore, MA, USA) according to the manufacturer's instructions. Cells were seeded into the upper inserts at 1×10^5^ cells/insert in William's E Medium with GlutaMAX. Outer wells were filled with William's E Medium with GlutaMAX. Cells were incubated at 37°C with 5% CO_2_ for 48 hours. The non-invading cells were removed. Cells that migrated through the gel insert to the lower surface were stained and quantified by colorimetric measurement at 560 nm.

### Chick embryo chorioallantoic membrane assay

Fertilised ISABrown layer strain chicken eggs (Roslin Institute Poultry Unit) were incubated in a humidified rotary incubator at 37°C. On day 3, a small window was opened in the shell after removal of 2-3 ml of albumin, to detach the CAM from the shell and to disclose the underlying CAM vessels. The window was sealed with tape and incubation was continued until day 7. On day 7, single cell suspensions of adherent cells and mammospheres were labelled with PKH26 (Sigma-Aldrich, MO, USA), a red fluorescent live cell membrane dye, according to manufacturer's instructions. Viable 10^5^ (n=4) cells were suspended in a 1:1 mixture of serum-free media:matrigel, and 25 μL was inoculated directly onto the CAM. The embryos were resealed and incubated without turning. At day 10, tumour growth and location were determined.

### Protein detection

Cells were lysed in urea lysis buffer (7 M urea, 0.1 M DTT, 0.05 % Triton X-100, 25 mM NaCl, 20 mM Hepes pH 7.5). Equal amounts of protein were separated by SDS polyacrylamide gel electrophoresis (SDS PAGE), transferred to Hybond-C nitrocellulose membrane (Amersham Pharmacia Biotech, Buckinghamshire, UK). Non-specific antibody binding was blocked by incubating membranes in 5 % milk/ 1 % β-glycerophosphate/ PBST for 1 hr at room temperature. β-glycerophosphate is a serine/threonine phosphatase inhibitor critical for the retention of phosphorylation modifications. Membranes were probed with appropriate primary antibody for 3 hr at room temperature or overnight at 4°C. Membranes were washed once in PBST. Specific antibody binding was detected by incubating membranes for 1 hr at room temperature with a secondary horseradish peroxidase (HRP) conjugated antibody diluted 1:1000 in 5 % milk/ 1% β-glycerophosphate/ PBST. Following three 15 min washes in PBST, membranes were treated with ECL chemiluminescent detection system (2 ml /blot, 1:1 ratio ECL I : ECL II) and protein bands were visualized by exposure to X-ray film (Kodak). Antibodies against Primary antibodies were β-actin, γ-H2AX, EGFR and phosphor-EGFR (Ser1047) (Abcam), β-catenin, E-cadherin and fibronectin (BD Biosciences), AKT, phospho-AKT (Ser473) and phospho-p53 (Ser15) (Cell Signaling), MDM2 (4B2) and p53 (DO1) (Moravain Biotechnology), phospho-EGFR (Tyr 1173) (SantaCruz Biotechnology). Secondary antibodies were HRP-conjugated rabbit anti-mouse IgG and swine anti-rabbit IgG (DakoCytomation).

### Subcellular proteome extraction

The Subcellular Proteome Fractionation Kit (Thermo Scientific) was used to extract proteins from mammalian cells according to their subcellular localisation. All fractions were stored at −70°C and analysed by immunoblotting (see above).

### RNA extraction and reverse transcription PCR analysis

Total cellular RNA was extracted using RNeasy^®^ kit (Qiagen, CA, USA) and RNA quality was determined by A_260_ measurement. Semi-quantitative RT-PCR analysis of mRNA expression of stem cell specific genes including *Oct4, Nanog* and *STAT3* was performed using HotStar *Taq* polymerase (Qiagen, CA, USA) and specific primers (Table 4.)

**Table 4:** Top biological functions of differentially expressed genes in J3T spheres compared to adherent cells (FDR = 0.005)

	p-Value	Number of molecules
**Diseases and Disorders**		
Genetic Disorder	2.66E-20– 9.88E-04	2087
Cancer	1.29E-17 – 9.49E-04	1254
Gastrointestinal Disease	1.29E-17 – 8.67E-04	1215
Developmental Disorder	2.84E-12 – 1.10E-11	142
Infectious Disease	3.30E-12 – 4.57E-04	466
**Molecular & Cellular Functions**		
Cell Cycle	1.40E-28 – 9.70E-04	544
Protein Synthesis	5.48E-20 – 5.91E-04	308
Cell Death	1.17E-19 – 9.57E-04	985
RNA Post-translational Modification	1.03E-17 – 7.49E-04	171
DNA Replication, Recombination and Repair	5.41E-17 – 9.25E-04	461
**Physiological System Development & Function**		
Organismal Survival	1.56E-09 – 4.94E-09	328
Organismal Development	9.61E-07 – 9.12E-04	267
Connective Tissue Development and Function	1.47E-05 – 5.88E-04	162
Tissue Development	1.47E-05 – 7.01E-04	151
Tumour Morphology	9.20E-05 – 9.20E-05	82

### Real-time quantitative PCR

Total RNA was reverse transcribed using the omniscript RT Kit (Qiagen, CA, USA) according to the manufacturer's instruction. Real-time PCR was performed on 50 ng of amplified RNA using a Stratagene Mx3000p qPCR system (Aligent, CA, USA), using the Platinum^®^ SYBR^®^ Green qPCR SuperMix-UDG according to manufacturer's instruction (Invitrogen, CA, USA). Relative gene expression levels of *TNFAIP6, EDNRA, NGR1* and *IL8* were obtained by normalization to the expression levels of housekeeping genes (*MRPS7*, *GAPDH*). Primer sequences are shown in Table 5.

### Gene expression profiling using cDNA microarrays

**Table 5:** Top ten upregulated genes in J3T spheres compared to adherent cells (FDR = 0.005)

Gene Symbol	Gene Name	Accession Number	Gene Ontology	Fold Change
EDNRA	endothelin receptor type A	P25101	G-protein coupled receptor	78.64
CXCR7	chemokine (C-X-C motif) receptor 7	P25106	G-protein coupled receptor	72.45
TNFAIP6	Tumor Necrosis Factor, Alpha-Induced Protein 6	P98066	extracellular matrix stability and cell migration	44.29
COL3A1	collagen, type III, alpha 1	P02461	Component of connective tissues	37.14
FAP	fibroblast activation protein, alpha	Q12884	Wound healing	28.68
LUM	lumican	P51884	Extra cellular matrix structural constituent	27.57
IGFBP2	insulin-like growth factor binding protein 2, 36kDa	P18065	Inhibition of IGF-mediated growth and development	27.13
LMCD1	LIM and cysteine-rich domains 1	Q9NZU5	Transcription corepressor activity	21.43
CDO1	cysteine dioxygenase, type I	Q16878	Cysteine dioxygenase activity	20.65
EGR1	early growth response 1	P18146	Transcriptional regulator	20.57

RNA was isolated from frozen cell pellets of untreated (0 Gy) and irradiated (5 Gy) J3T spheres and adherent cells, with TriReagent (Sigma-Aldrich, MO, USA) according to manufacturer's instruction. Four independent replicates were used for each sample. Total RNA quality was determined by Bioanalyser (Agilent, CA, USA) before further manipulation. Complementary RNA preparation and hybridization were performed by ARK-Genomics (Edinburgh, UK) using Affymetrix GeneChip® Canine Genome 2.0 Array (42,800 probe sets). Basic data analysis was performed using the Partek Genomics Suite (Partek Inc, MO, USA). Pathway analysis was performed using Ingenuity Pathway Analysis (IPA, Ingenuity systems; https://www.analysis.ingenuity.com). Genes from the dataset that met the log ratio cut-off of 1.5 were considered for the analysis. To identify the most relevant canonical pathways, we selected those that were statistically significant with a *p* value < 0.005. All microarray data has been submitted to the NCBI Gene Expression Omnibus database.

### Statistical analysis

Data were expressed as a mean ±SD. Statistical analysis was performed with Minitab® statistical software (PA, USA) using analysis of variance and student's t test or mann-whitney test. The criterion for significance was p < 0.05 for all comparisons.

## CONCLUSIONS

In summary, our findings demonstrate that CSCs exist in the canine glioma cell line, J3T and the human glioma cell line, LN18. These cells are more invasive, more tumourigenic and more resistant to chemotherapy and irradiation compared with non-CSC glioma cells. Global gene expression analysis has demonstrated that the gene expression profiles of these subpopulations of glioma cells significantly differ, and we have consequently shown differences in the p53 and EGFR signaling pathways. Activators of the EGFR pathway were over-expressed in CSCs compared to non-CSCs, and we subsequently showed that CSCs were more sensitive to the effect of gefitinib, which enhanced radiosensitivity and increased cytotoxicity.

## References

[1] Dickinson PJ, LeCouteur RA, Higgins RJ, Bringas JR, Larson RF, Yamashita Y, Krauze MT, Forsayeth J, Noble CO, Drummond DC, Kirpotin DB, Park JW, Berger MS, Bankiewicz KS (2010). Canine spontaneous glioma: a translational model system for convection-enhanced delivery. Neuro Oncol.

[2] Herranz C, Fernandez F, Martin-Ibanez R, Blasco E, Crespo E, De la Fuente C, Anor S, Rabanal RM, Canals JM, Pumarola M (2016). Spontaneously arising canine glioma as a potential model for human glioma. J Comp Pathol.

[3] Lipsitz D, Higgins RJ, Kortz GD, Dickinson PJ, Bollen AW, Naydan DK, LeCouteur RA (2003). Glioblastoma multiforme: clinical findings, magnetic resonance imaging, and pathology in five dogs. Vet Pathol.

[4] deSouza RM, Shaweis H, Han C, Sivasubramiam V, Brazil L, Beaney R, Sadler G, Al-Sarraj S, Hampton T, Logan J, Hurwitz V, Bhangoo R, Gullan R, Ashkan K (2016). Has the survival of patients with glioblastoma changed over the years?. Br J Cancer.

[5] Chen L, Zhang Y, Yang J, Hagan JP, Li M (2013). Vertebrate animal models of glioma: understanding the mechanisms and developing new therapies. Biochim Biophys Acta.

[6] Pang LY, Argyle DJ (2009). Using naturally occurring tumours in dogs and cats to study telomerase and cancer stem cell biology. Biochim Biophys Acta.

[7] Bergkvist GT, Yool DA (2011). Epidermal growth factor receptor as a therapeutic target in veterinary oncology. Vet Comp Oncol.

[8] Dirks PB (2008). Brain tumor stem cells: bringing order to the chaos of brain cancer. J Clin Oncol.

[9] Pang LY, Argyle DJ (2015). The evolving cancer stem cell paradigm: implications in veterinary oncology. Vet J.

[10] Galli R, Binda E, Orfanelli U, Cipelletti B, Gritti A, De Vitis S, Fiocco R, Foroni C, Dimeco F, Vescovi A (2004). Isolation and characterization of tumorigenic, stem-like neural precursors from human glioblastoma. Cancer Res.

[11] Hemmati HD, Nakano I, Lazareff JA, Masterman-Smith M, Geschwind DH, Bronner-Fraser M, Kornblum HI (2003). Cancerous stem cells can arise from pediatric brain tumors. Proc Natl Acad Sci U S A.

[12] Singh SK, Clarke ID, Terasaki M, Bonn VE, Hawkins C, Squire J, Dirks PB (2003). Identification of a cancer stem cell in human brain tumors. Cancer Res.

[13] Singh SK, Hawkins C, Clarke ID, Squire JA, Bayani J, Hide T, Henkelman RM, Cusimano MD, Dirks PB (2004). Identification of human brain tumour initiating cells. Nature.

[14] Stoica G, Lungu G, Martini-Stoica H, Waghela S, Levine J, Smith R (2009). Identification of cancer stem cells in dog glioblastoma. Vet Pathol.

[15] Yuan X, Curtin J, Xiong Y, Liu G, Waschsmann-Hogiu S, Farkas DL, Black KL, Yu JS (2004). Isolation of cancer stem cells from adult glioblastoma multiforme. Oncogene.

[16] Hatanpaa KJ, Burma S, Zhao D, Habib AA (2010). Epidermal growth factor receptor in glioma: signal transduction, neuropathology, imaging, and radioresistance. Neoplasia.

[17] Nicholas MK, Lukas RV, Jafri NF, Faoro L, Salgia R (2006). Epidermal growth factor receptor - mediated signal transduction in the development and therapy of gliomas. Clin Cancer Res.

[18] Roth P, Weller M (2014). Challenges to targeting epidermal growth factor receptor in glioblastoma: escape mechanisms and combinatorial treatment strategies. Neuro Oncol.

[19] Tebbutt N, Pedersen MW, Johns TG (2013). Targeting the ERBB family in cancer: couples therapy. Nat Rev Cancer.

[20] Azuaje F, Tiemann K, Niclou SP (2015). Therapeutic control and resistance of the EGFR-driven signaling network in glioblastoma. Cell Commun Signal.

[21] Roberts PJ, Der CJ (2007). Targeting the Raf-MEK-ERK mitogen-activated protein kinase cascade for the treatment of cancer. Oncogene.

[22] Bandyopadhyay D, Mandal M, Adam L, Mendelsohn J, Kumar R (1998). Physical interaction between epidermal growth factor receptor and DNA-dependent protein kinase in mammalian cells. J Biol Chem.

[23] Kang KB, Zhu C, Wong YL, Gao Q, Ty A, Wong MC (2012). Gefitinib radiosensitizes stem-like glioma cells: inhibition of epidermal growth factor receptor-Akt-DNA-PK signaling, accompanied by inhibition of DNA double-strand break repair. Int J Radiat Oncol Biol Phys.

[24] Christiansen JJ, Rajasekaran AK (2006). Reassessing epithelial to mesenchymal transition as a prerequisite for carcinoma invasion and metastasis. Cancer Res.

[25] Thiery JP (2003). Epithelial-mesenchymal transitions in development and pathologies. Curr Opin Cell Biol.

[26] Kastan MB, Onyekwere O, Sidransky D, Vogelstein B, Craig RW (1991). Participation of p53 protein in the cellular response to DNA damage. Cancer Res.

[27] Rogakou EP, Pilch DR, Orr AH, Ivanova VS, Bonner WM (1998). DNA double-stranded breaks induce histone H2AX phosphorylation on serine 139. J Biol Chem.

[28] Hupp TR, Lane DP, Ball KL (2000). Strategies for manipulating the p53 pathway in the treatment of human cancer. Biochem J.

[29] Ke C, Tran K, Chen Y, Di Donato AT, Yu L, Hu Y, Linskey ME, Wang PH, Limoli CL, Zhou YH (2014). Linking differential radiation responses to glioma heterogeneity. Oncotarget.

[30] Mazzoleni S, Politi LS, Pala M, Cominelli M, Franzin A, Sergi Sergi L, Falini A, De Palma M, Bulfone A, Poliani PL, Galli R (2010). Epidermal growth factor receptor expression identifies functionally and molecularly distinct tumor-initiating cells in human glioblastoma multiforme and is required for gliomagenesis. Cancer Res.

[31] Yin AH, Miraglia S, Zanjani ED, Almeida-Porada G, Ogawa M, Leary AG, Olweus J, Kearney J, Buck DW (1997). AC133, a novel marker for human hematopoietic stem and progenitor cells. Blood.

[32] Uchida N, Buck DW, He D, Reitsma MJ, Masek M, Phan TV, Tsukamoto AS, Gage FH, Weissman IL (2000). Direct isolation of human central nervous system stem cells. Proc Natl Acad Sci U S A.

[33] Beier D, Hau P, Proescholdt M, Lohmeier A, Wischhusen J, Oefner PJ, Aigner L, Brawanski A, Bogdahn U, Beier CP (2007). CD133(+) and CD133(−) glioblastoma-derived cancer stem cells show differential growth characteristics and molecular profiles. Cancer Res.

[34] Ogden AT, Waziri AE, Lochhead RA, Fusco D, Lopez K, Ellis JA, Kang J, Assanah M, McKhann GM, Sisti MB, McCormick PC, Canoll P, Bruce JN (2008). Identification of A2B5+CD133- tumor-initiating cells in adult human gliomas. Neurosurgery.

[35] Joo KM, Kim SY, Jin X, Song SY, Kong DS, Lee JI, Jeon JW, Kim MH, Kang BG, Jung Y, Jin J, Hong SC, Park WY (2008). Clinical and biological implications of CD133-positive and CD133-negative cells in glioblastomas. Lab Invest.

[36] Brescia P, Ortensi B, Fornasari L, Levi D, Broggi G, Pelicci G (2013). CD133 is essential for glioblastoma stem cell maintenance. Stem Cells.

[37] Menendez S, Camus S, Izpisua Belmonte JC (2010). p53: guardian of reprogramming. Cell Cycle.

[38] Xu Y (2005). A new role for p53 in maintaining genetic stability in embryonic stem cells. Cell Cycle.

[39] Weissbein U, Benvenisty N, Ben-David U (2014). Quality control: genome maintenance in pluripotent stem cells. J Cell Biol.

[40] Wang Y, Chen J, Li Q, Wang H, Liu G, Jing Q, Shen B (2011). Identifying novel prostate cancer associated pathways based on integrative microarray data analysis. Comput Biol Chem.

[41] Salazar N, Munoz D, Kallifatidis G, Singh RK, Jorda M, Lokeshwar BL (2014). The chemokine receptor CXCR7 interacts with EGFR to promote breast cancer cell proliferation. Mol Cancer.

[42] Singh RK, Lokeshwar BL (2011). The IL-8-regulated chemokine receptor CXCR7 stimulates EGFR signaling to promote prostate cancer growth. Cancer Res.

[43] Chua CY, Liu Y, Granberg KJ, Hu L, Haapasalo H, Annala MJ, Cogdell DE, Verploegen M, Moore LM, Fuller GN, Nykter M, Cavenee WK, Zhang W (2016). IGFBP2 potentiates nuclear EGFR-STAT3 signaling. Oncogene.

[44] Nishi H, Nishi KH, Johnson AC (2002). Early growth response-1 gene mediates up-regulation of epidermal growth factor receptor expression during hypoxia. Cancer Res.

[45] Holohan C, Van Schaeybroeck S, Longley DB, Johnston PG (2013). Cancer drug resistance: an evolving paradigm. Nat Rev Cancer.

[46] Higgins RJ, Dickinson PJ, LeCouteur RA, Bollen AW, Wang H, Wang H, Corely LJ, Moore LM, Zang W, Fuller GN (2010). Spontaneous canine gliomas: overexpression of EGFR, PDGFRalpha and IGFBP2 demonstrated by tissue microarray immunophenotyping. J Neurooncol.

[47] Dickinson PJ, Roberts BN, Higgins RJ, Leutenegger CM, Bollen AW, Kass PH, LeCouteur RA (2006). Expression of receptor tyrosine kinases VEGFR-1 (FLT-1), VEGFR-2 (KDR), EGFR-1, PDGFRalpha and c-Met in canine primary brain tumours. Vet Comp Oncol.

[48] Bernier J, Schneider D (2007). Cetuximab combined with radiotherapy: an alternative to chemoradiotherapy for patients with locally advanced squamous cell carcinomas of the head and neck?. Eur J Cancer.

[49] Thariat J, Yildirim G, Mason KA, Garden AS, Milas L, Ang KK (2007). Combination of radiotherapy with EGFR antagonists for head and neck carcinoma. Int J Clin Oncol.

[50] Mellinghoff IK, Wang MY, Vivanco I, Haas-Kogan DA, Zhu S, Dia EQ, Lu KV, Yoshimoto K, Huang JH, Chute DJ, Riggs BL, Horvath S, Liau LM (2005). Molecular determinants of the response of glioblastomas to EGFR kinase inhibitors. N Engl J Med.

[51] Munoz JL, Rodriguez-Cruz V, Greco SJ, Ramkissoon SH, Ligon KL, Rameshwar P (2014). Temozolomide resistance in glioblastoma cells occurs partly through epidermal growth factor receptor-mediated induction of connexin 43. Cell Death Dis.

[52] Vivanco I, Robins HI, Rohle D, Campos C, Grommes C, Nghiemphu PL, Kubek S, Oldrini B, Chheda MG, Yannuzzi N, Tao H, Zhu S, Iwanami A (2012). Differential sensitivity of glioma- versus lung cancer-specific EGFR mutations to EGFR kinase inhibitors. Cancer Discov.

[53] Schmoll HJ, Van Cutsem E, Stein A, Valentini V, Glimelius B, Haustermans K, Nordlinger B, van de Velde CJ, Balmana J, Regula J, Nagtegaal ID, Beets-Tan RG, Arnold D (2012). ESMO Consensus Guidelines for management of patients with colon and rectal cancer. A personalized approach to clinical decision making. Ann Oncol.

[54] Tol J, Punt CJ (2010). Monoclonal antibodies in the treatment of metastatic colorectal cancer: a review. Clin Ther.

[55] Cardoso F, Fallowfield L, Costa A, Castiglione M, Senkus E, ESMO Guidelines Working Group (2011). Locally recurrent or metastatic breast cancer: ESMO Clinical Practice Guidelines for diagnosis, treatment and follow-up. Ann Oncol.

[56] Harris CA, Ward RL, Dobbins TA, Drew AK, Pearson S (2011). The efficacy of HER2-targeted agents in metastatic breast cancer: a meta-analysis. Ann Oncol.

[57] Reardon DA, Groves MD, Wen PY, Nabors L, Mikkelsen T, Rosenfeld S, Raizer J, Barriuso J, McLendon RE, Suttle AB, Ma B, Curtis CM, Dar MM, de Bono J (2013). A phase I/II trial of pazopanib in combination with lapatinib in adult patients with relapsed malignant glioma. Clin Cancer Res.

[58] Reardon DA, Nabors LB, Mason WP, Perry JR, Shapiro W, Kavan P, Mathieu D, Phuphanich S, Cseh A, Fu Y, Cong J, Wind S, Eisenstat DD, BI 1200 36 Trial Group, Canadian Brain Tumour Consortium (2015). Phase I/randomized phase II study of afatinib, an irreversible ErbB family blocker, with or without protracted temozolomide in adults with recurrent glioblastoma. Neuro Oncol.

[59] Wen PY, Chang SM, Lamborn KR, Kuhn JG, Norden AD, Cloughesy TF, Robins HI, Lieberman FS, Gilbert MR, Mehta MP, Drappatz J, Groves MD, Santagata S (2014). Phase I/II study of erlotinib and temsirolimus for patients with recurrent malignant gliomas: North American Brain Tumor Consortium Trial 04-02. Neuro Oncol.

[60] Geoerger B, Gaspar N, Opolon P, Morizet J, Devanz P, Lecluse Y, Valent A, Lacroix L, Grill J, Vassal G (2008). EGFR tyrosine kinase inhibition radiosensitizes and induces apoptosis in malignant glioma and childhood ependymoma xenografts. Int J Cancer.

[61] Sarkaria JN, Carlson BL, Schroeder MA, Grogan P, Brown PD, Giannini C, Ballman KV, Kitange GJ, Guha A, Pandita A, James CD (2006). Use of an orthotopic xenograft model for assessing the effect of epidermal growth factor receptor amplification on glioblastoma radiation response. Clin Cancer Res.

[62] Solomon B, Hagekyriakou J, Trivett MK, Stacker SA, McArthur GA, Cullinane C (2003). EGFR blockade with ZD1839 (“Iressa”) potentiates the antitumor effects of single and multiple fractions of ionizing radiation in human A431 squamous cell carcinoma. Epidermal growth factor receptor. Int J Radiat Oncol Biol Phys.

[63] Stea B, Falsey R, Kislin K, Patel J, Glanzberg H, Carey S, Ambrad AA, Meuillet EJ, Martinez JD (2003). Time and dose-dependent radiosensitization of the glioblastoma multiforme U251 cells by the EGF receptor tyrosine kinase inhibitor ZD1839 (‘Iressa’). Cancer Lett.

[64] Chakravarti A, Wang M, Robins HI, Lautenschlaeger T, Curran WJ, Brachman DG, Schultz CJ, Choucair A, Dolled-Filhart M, Christiansen J, Gustavson M, Molinaro A, Mischel P (2013). RTOG 0211: a phase 1/2 study of radiation therapy with concurrent gefitinib for newly diagnosed glioblastoma patients. Int J Radiat Oncol Biol Phys.

[65] Prados MD, Chang SM, Butowski N, DeBoer R, Parvataneni R, Carliner H, Kabuubi P, Ayers-Ringler J, Rabbitt J, Page M, Fedoroff A, Sneed PK, Berger MS (2009). Phase II study of erlotinib plus temozolomide during and after radiation therapy in patients with newly diagnosed glioblastoma multiforme or gliosarcoma. J Clin Oncol.

[66] Ekstrand AJ, Longo N, Hamid ML, Olson JJ, Liu L, Collins VP, James CD (1994). Functional characterization of an EGF receptor with a truncated extracellular domain expressed in glioblastomas with EGFR gene amplification. Oncogene.

[67] Inoue S, Ichikawa T, Kurozumi K, Maruo T, Onishi M, Yoshida K, Fujii K, Kambara H, Chiocca EA, Date I (2012). Novel animal glioma models that separately exhibit two different invasive and angiogenic phenotypes of human glioblastomas. World Neurosurg.

[68] Rainov NG, Koch S, Sena-Esteves M, Berens ME (2000). Characterization of a canine glioma cell line as related to established experimental brain tumor models. J Neuropathol Exp Neurol.

[69] Diserens AC, de Tribolet N, Martin-Achard A, Gaide AC, Schnegg JF, Carrel S (1981). Characterization of an established human malignant glioma cell line: LN-18. Acta Neuropathol.

